# Sustained Reductions of Bay Area CO_2_ Emissions 2018–2022

**DOI:** 10.1021/acs.est.3c09642

**Published:** 2024-04-04

**Authors:** Naomi G. Asimow, Alexander J. Turner, Ronald C. Cohen

**Affiliations:** † Department of Earth and Planetary Science, University of California, Berkeley, Berkeley, California 94720, United States; ‡ College of Chemistry, University of California, Berkeley, Berkeley, California 94720, United States

**Keywords:** greenhouse gas emissions, climate change, inverse modeling, carbon dioxide, sensor networks

## Abstract

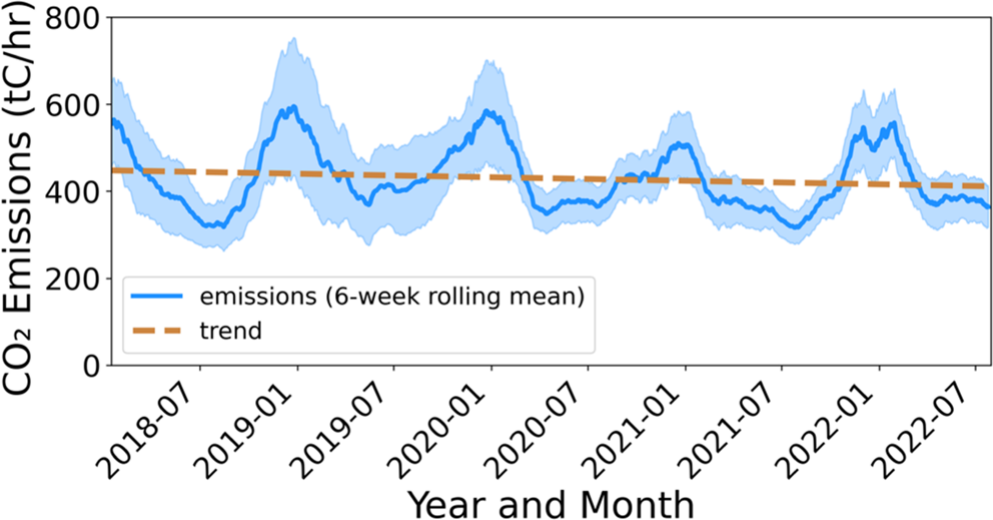

Cities represent a significant and growing portion of global carbon dioxide (CO_2_) emissions. Quantifying urban emissions and trends over time is needed to evaluate the efficacy of policy targeting emission reductions as well as to understand more fundamental questions about the urban biosphere. A number of approaches have been proposed to measure, report, and verify (MRV) changes in urban CO_2_ emissions. Here we show that a modest capital cost, spatially dense network of sensors, the Berkeley Environmental Air Quality and CO_2_ Network (BEACO_2_N), in combination with Bayesian inversions, result in a synthesis of measured CO_2_ concentrations and meteorology to yield an improved estimate of CO_2_ emissions and provide a cost-effective and accurate assessment of CO_2_ emissions trends over time. We describe nearly 5 years of continuous CO_2_ observations (2018–2022) in a midsized urban region (the San Francisco Bay Area). These observed concentrations constrain a Bayesian inversion that indicates the interannual trend in urban CO_2_ emissions in the region has been a modest decrease at a rate of 1.8 ± 0.3%/year. We interpret this decrease as primarily due to passenger vehicle electrification, reducing on-road emissions at a rate of 2.6 ± 0.7%/year.

## Abstract 2

This study uses a dense sensor network of CO_2_ observations to constrain 5 years of CO_2_ emissions in the San Francisco Bay Area.

## 1 Introduction

Reversing global trends in carbon dioxide (CO_2_) emissions represents one of the greatest challenges facing humankind today. CO_2_ is the most important greenhouse gas (GHG) and limiting warming to 1.5 or 2 °C demands achieving global net-zero CO_2_ emissions by the early 2050s or 2070s, respectively.^1^ These pathways require aggressive changes to the global energy infrastructure. Cities represent over 70% of global CO_2_ emissions, a fraction that is projected to grow as people continue to migrate into urban areas. Many cities have set net-zero greenhouse gas or other ambitious CO_2_ targets for the coming decades, such as Boston (carbon-neutral by 2050), Copenhagen (carbon-neutral by 2025), New York and London (80% reductions by 2050), and San Francisco (net-zero by 2040).^2^ Carbon neutrality goals from different cities may vary significantly in their definitions of carbon emissions (i.e., accounting for only scope 1 emissions or also scope 2 or 3) and their definition of neutrality (i.e., legitimacy of purchasing of carbon offsets to reduce net emissions). Consortiums of city governments are also emerging to help compare goals between cities and develop common accounting practices. These groups include C40 cities,^3^ Local Governments for Sustainability,^4^ and Carbon Neutral Cities Alliance.^2^

As cities set greenhouse gas reduction targets, there is an emerging need to support them with strategies to monitor and quantify urban CO_2_ emissions. Historically, emissions have been quantified and reported through bottom-up accounting strategies based primarily on economic activity data or emissions inventories. However, such strategies have been shown to have large uncertainties,^5^ more often in the direction of under-reporting emissions.^6^

Given the known inadequacies of activity-based emissions reporting, there is significant interest in using atmospheric measurements to constrain emissions of CO_2_ and other species.^7^ Measurements of the CO_2_ concentrations can be related to emissions by identifying the location of emissions that are responsible for that concentration. If a background CO_2_ concentration outside the domain of interest can be defined, a Lagrangian back trajectory model can be used to compute surface influence footprints that quantify the contribution of CO_2_ emissions at each location in the domain to a measurement of the CO_2_ concentration. Combining the footprints with observations of concentrations over time allows a Bayesian update on an a priori emissions inventory created from bottom-up accounting methods. The resulting emissions inventory is an optimized combination of our knowledge of activity and observed CO_2_. Bayesian inversions have been applied to cities with CO_2_ observations that are as accurate as modern technology allows; this method has shown success in reducing the uncertainty in emissions estimates compared to traditional emission inventories, especially for monthly or yearly estimates.^8−13^ The measurements in these networks require significant capital investment at each location, and maintaining the highest accuracy and precision is labor-intensive.

There are a number of sources of uncertainty when using atmospheric inversions to constrain emissions, for which results can be quite sensitive. Instrument error results from the sensitivity and accuracy of the observational instrument (and associated calibration and maintenance procedures) used to measure atmospheric concentrations. Model errors can result from uncertainty in background concentrations, prior biospheric fluxes, prior anthropogenic fluxes, wind direction, and planetary boundary layer heights (PBLH), particularly during the night time.^14−17^ Representation errors result from the numerical discretization of the model (i.e., space and time resolution that is not representative of measurement scales); for instance, a 1 × 1 km pixel of a model may not be representative of the measurement within that pixel. Generally, errors in the instrument, model, and representation are assumed to be uncorrelated and thus additive (where the sum is referred to as mismatch error), so error in any of these terms can dominate the mismatch error and thus the uncertainty in the resulting posterior flux estimate. Note that throughout this paper, the terms “emission” and “flux” are used interchangeably, referring to the mass of carbon exchanged per unit area over time (measured in units of mass/area/time). When aggregating emissions for larger regions, we omit the per area units, presenting certain results in mass/time units.

There have only been a few attempts to provide multiyear and observationally constrained CO_2_ emission inventories, though many other publications have applied this methodology over shorter time scales. We note the study in Los Angeles, CA covering the period of 2006–2013 which showed an emission reduction of 10% during the 2008–2010 recession.^18^ Emissions were estimated from 2012 to 2015 for the Indianapolis region^19^ and from 2013 to 2014 in the Boston region.^20^ A recent multiyear inversion in Paris showed a decreasing trend of around 2%/year in CO_2_ emissions from 2016 to 2021.^21^ A comparative analysis of Los Angeles and Washington, DC/Baltimore Metropolitan areas conducted inversions in these two cities for 2018–2020 to quantify COVID-19-related emissions reductions.^22^ A study of the Salt Lake Valley used a multiyear CO_2_ inversion to examine the relationship between emissions and urban population density.^23^ Each of these studies took the approach of using a relatively small (range: 2–13) number of high-accuracy (∼0.1 ppm) monitoring sites to constrain CO_2_ fluxes in an urban area.

The San Francisco Bay Area is an interesting policy laboratory for GHG reduction. It contains numerous independent cities and counties as well as a variety of overlapping regional metropolitan authorities and agencies (e.g., transportation agencies, air quality management board, and air resources board). Some California cities have matched their stated net-zero targets to that of the State of California’s net-zero by 2045 policy, such as Berkeley^24^ and Oakland^25^ but some seek faster reductions, such as San Francisco, which has accelerated their net-zero goal to 2040.^26^ Still, other Bay Area cities have yet to pass resolutions or publish plans with net-zero targets. Further, the San Francisco Bay Area is an interesting region to assess the potential for analyses of spatially dense sensor networks to provide cost-effective multiyear constraints on emission trends and policy efficacy. The region has complex topography (terrain height ranging from sea level to about 500 m) and diverse land uses. It has emissions that are dominated by transportation but include significant heavy industry and also heating from natural gas. A dense sensor network is well poised to capture the heterogeneous CO_2_ concentrations in this diverse area. Additionally, models that effectively constrain emissions in the Bay Area have the potential for successful translation to other urban areas.

**Figure 1:**
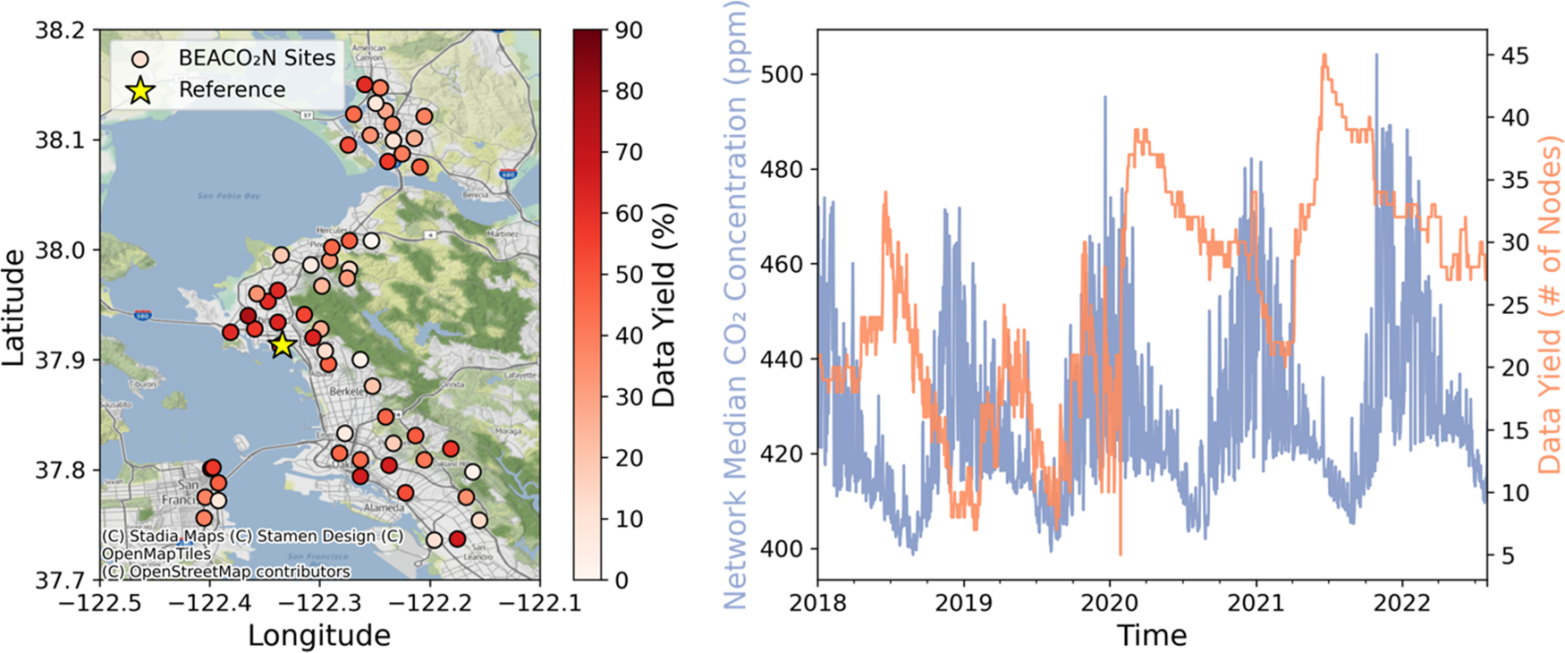
Map and time series of BEACO_2_N network coverage during the study period. (Left) Percentage of hours (out of 5 years) with usable measurements after quality review for each site. (Right) Daily median CO_2_ concentration across all sites (purple) and the number of sites with usable data (orange). Background map credits: © Stadia Maps (stadiamaps.com), © Stamen Design (stamen.com), © OpenMapTiles (openmaptiles.org), and © OpenStreetMap (openstreetmap.org/copyright).

Spatially dense, frequent CO_2_ measurements for a region of the Bay Area are available from the Berkeley Environmental Air Quality and CO_2_ Network (BEACO_2_N).^27^ This network was designed with a target 2 km node spacing, 1 ppm hourly measurement uncertainty for CO_2_, and 5 s sampling frequency in order to have sensitivity to local emissions. Delaria et al. analyzed site-to-site variation in the BEACO_2_N network as an upper bound on sensor accuracy and found that the network achieves a total error of 1.6 ppm or less, close to our 1 ppm goal.^28^ The network also includes measurements of air quality-relevant species (CO, NO, NO_2_, O_3_, and PM_2.5_) at every node. The BEACO_2_N project also maintains a reference site at the UC Berkeley Richmond Field Station with calibrated, reference quality measurements of CO_2_, CO, CH_4_, NO, NO_2_, O_3_, and size-resolved measurements of PM. Most of the BEACO_2_N nodes are located on school rooftops, with inlet heights ranging from 1 to 120 m above ground level (median height = 5 m AGL). During the study period (January 2018–July 2022), sensors were located at the 57 sites shown in Figure 1a. This network provides tremendous additional monitoring capacity, as the EPA operates only 7 air quality monitoring stations in the BEACO_2_N region. Importantly, these EPA sites do not monitor the level of CO_2_. Our data set provides the first opportunity to use ground-based observations to constrain trends in Bay Area CO_2_ emissions.

While other multiyear urban CO_2_ inversion studies have utilized fewer observation sites with higher sensor accuracy, our approach uses a higher number of lower-cost, moderate-accuracy sensors. Turner et al.^29^ examined the uncertainty in the derived emissions using simulated observations from potential Bay Area CO_2_ measurement networks with varied sensory precision and node number. That study showed that a 25 site, 1 ppm accuracy, BEACO_2_N-like network (smaller than the current BEACO_2_N network but with the same 2 km spacing) gave similar error in posterior emissions estimates to a 3 site, 0.1 ppm accuracy network (of comparable total capital cost). Turner et al.^30^ demonstrated the use of BEACO_2_N measurements in an atmospheric inversion framework to quantify the drop in emissions during the COVID-19 shelter-in-place order (March 2020–May 2020), a period of notable emissions shifts globally.^31^

In this paper, we extend and modify the methodology of Turner et al.^30^ by combining almost 5 years of CO_2_ observations from BEACO_2_N with a Bayesian inversion to quantify interannual trends in Bay Area scope 1 CO_2_ emissions. Our previous study treated the background concentration as a known value and used the spatial distribution of emissions to allocate emissions to different sectors using the prior distribution of emissions. We update this methodology to solve dynamically for background concentrations and employ a linear regression method to examine emissions by sector. Extending the analysis to a 5-year time period allows us to examine interannual trends. We find a decreasing emissions trend of 1.8 ± 0.3%/year over the region from 2018 to 2022. We use measured traffic data and utility natural gas distribution data to decompose the sectoral contributions to the total emissions and understand seasonality and factors driving interannual trends in emissions.

## 2 Methods

For a process flow diagram of the methods and data sets used, refer to Figure S1.

### 2.1 BEACO_2_N Observations

Measurements of ambient CO_2_ concentrations were made at BEACO_2_N locations throughout the San Francisco Bay Area using a Vaisala CARBOCAP Carbon Dioxide Probe GMP343. Measurements were recorded approximately every 5 s.

Concentrations were averaged hourly and calibrated following procedures outlined in Delaria et al.^28^ that account for individual sensor temperature dependence. Sensors are offset corrected to measurements from a reference-grade Picarro G2301 gas concentration analyzer located at UC Berkeley’s Richmond Field Station Campus (yellow star in Figure 1). The Picarro G2301 has reported 5 min instrument precision of <25 ppb and reported maximum monthly drift of 500 ppb.^32^ The instrument calibration was checked approximately every 3 months using a reference gas canister. No significant drift was observed. Calibrated BEACO_2_N data were manually inspected for quality, and time periods with unreliable data were removed before further analysis. Figure 1 shows the data coverage after quality review. The full study data set extends from January 2018 through the end of July 2022. The median CO_2_ concentration across all sites, shown in Figure 1(right), describes the regional CO_2_ concentration changes experienced in this time period. Concentrations are higher in the winter and lower in the summer. In winter, the region also frequently experiences large enhancements over background concentrations. 15.4% of the collected data did not pass quality assurance: 7.4% due to malfunction of the colocated temperature/pressure/humidity sensor and 8% due to malfunction of the CO_2_ sensor or other suspicious CO_2_ signals (such as cases where extreme hyperlocal enhancements from building exhaust were identified).

### 2.2 Prior Emissions Inventory

The prior emissions used are a 1 km resolution hourly bottom-up inventory, which incorporates traffic CO_2_ emissions from a fuel-based inventory for vehicle emissions (FIVE), The Bay Area Air Quality Management District (BAAQMD) 2010 report of large point sources, and county-level residential fuel usage (from BAAQMD) scaled to block-level population data from the 2010 US census.^29,30,33,34^ The prior biosphere fluxes are derived from measurements of solar-induced fluorescence (SIF) from The TROPOspheric Monitoring Instrument (TROPOMI), scaled using the Solar-Induced Fluorescence to Gross Primary Production (SIF-GPP) relationships described by Turner et al.^35^ The prior inventory has CO_2_ emissions in the region of influence that increase slightly each year from 496 to 518 tC/h over 2018–2022. The prior inventory includes diurnal traffic flow patterns (different for weekdays and weekends) that do not vary with season or year. There is no diurnal or seasonal cycle in the point source emissions. Annual scaling is applied to the point source emissions to match Mangat et al.^34^ The prior data do not vary with season due to the temporal resolution of the inputs (the BAAQMD inventory provides annual estimates with no monthly breakdown). No information related to changing emissions due to activity changes during the COVID-19 shelter-in-place (March 16, 2020–May 4, 2020) was included in the prior.

### 2.3 Prior Background Concentrations

For prior background concentrations, we used OCO-2 GEOS (Goddard Earth Observing System) L3 assimilated data set with global coverage, 3 h temporal resolution, 0.5 degree horizontal spatial resolution, and 72 vertical levels.^36^ The values at each of the centers of the four domain edges were selected (from the lowest vertical level of the model to correspond to near-ground concentrations), and a simple quadratic interpolation was used to resample the resolution to hourly at each edge. Prior values are shown in Figure S9a.

### 2.4 Computation of Footprints

For each BEACO_2_N observation (hourly mean concentration at each site), a surface influence footprint is calculated using the Stochastic Time-Inverted Lagrangian Transport Model (STILT)^37,38^ with meteorology from NOAA’s High Resolution Rapid Refresh (HRRR) product.^39^ HRRR has a 3 km spatial resolution and hourly temporal resolution. For each BEACO_2_N measurement location and time, 1000 hypothetical particles were advected backward in time and space for 72 h (or until all particles left the STILT domain extending from 36°N to 40°N and 125°W to 120°W). The standard assumption in the STILT model is that particles within one-half the height of the boundary layer are representative of trajectories of emissions from the surface that reach the receptor.^40^ STILT sums the particles within half of the planetary boundary layer height to compute surface influence footprints in units of parts per million (parts per million) (μmol/m^2^/s) at 1 km resolution. STILT computes PBLH from HRRR meteorology using a modified Richardson number.^41^ The footprints represent the expected enhancement in observed CO_2_ concentration due to a mass of emitted CO_2_ from each grid pixel (a flux). The forward model, eq 1, uses footprints to relate observed CO_2_ concentrations to emissions:
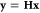
1where **y** is an *n* × 1 column vector of concentrations in units of ppm, the state vector **x** is an *m* × 1 column vector of surface fluxes in units of μmol/m^2^/s, and **H** is an *n* × *m* matrix of STILT footprints (in units of ppm/(μmol/m^2^/s)) where each row represents (for one observation in **y**) the sensitivity of an observation to each of the fluxes in **x.** The product of footprints and fluxes (**Hx**) gives the concentration enhancement resulting from emissions, not the total concentration. As such, it is typical to formulate **y** as a vector of concentration enhancements by subtracting a background concentration from each observation. However, in this study, we invert for the background concentrations directly, rather than treating the background as known. We therefore formulate **H** and **x** with additional parameters relating to the backgrounds, as described in additional detail in Section 2.5 and Text S1. As such, our formulation of **y** contains the values of total concentrations.

### 2.5 Inversion Framework

We invert both the fluxes within the specified domain and the background concentration at the domain edges. The footprints were coupled with the emissions prior and an estimate of the background to solve for posterior fluxes and backgrounds following eq 2:
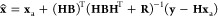
2where **x̂** is a vector of the posterior fluxes at each hour and grid-cell, and the background concentrations at each hour and each of the four domain edges, **x**_a_ contains the prior value of the fluxes (emissions inventory) and the prior background concentrations, **H** is the operator that connects the observations to emissions, combining the HRRR-STILT footprints and indicator values (0 or 1) for which background concentration to use, **B** is a prior error covariance matrix, **R** is the model-data mismatch error covariance matrix, and **y** is the BEACO_2_N measurements. This equation is derived from assuming Gaussian distributions of errors and solving for the probability density function *P*(**x**|**y**), where **x̂** is the expected value of the probability density function.^42^ For computational efficiency we express the prior error covariance matrix **B** as a Kronecker product of a spatial prior error covariance matrix and a temporal prior error covariance matrix, as described by Yadav and Michalak, 2013.^43^ We solve eq 2 to generate posterior fluxes once for each day of the study period using 96 h overlapping windows. See Text S1 for additional details on the inversion framework.

### 2.6 Determination of Influence Region

We do not have sensitivity to all emissions within the 157 km × 127 km domain of the inversion. Computation of the diagonal of the averaging kernel matrix is one method to define the region of interest, but this matrix has dimension *m* × *m*, and thus computing it directly is typically computationally intractable. In lieu of constructing the full matrix, we calculate the cumulative influence of the footprints in the region and define the top 40th percentile as the region to which we are sensitive (influence region). Posterior fluxes were analyzed only within the influence region containing 40% of the cumulative footprint surface influence. See Text S2 for additional details about the influence region.

### 2.7 Traffic Flow and Natural Gas Data Sets

Traffic flow data were obtained from the Caltrans Performance Measurement System (PeMS).^44^ Data for 693 PeMS observation sites within the BEACO_2_N region of influence as of 2018 were included in our assessments. We calculated vehicle miles traveled (VMT) as the product of the vehicle count at each PeMS site and the segment length to the next PeMS site. At each hour, we summed the VMT across any PeMS sites in the BEACO_2_N region of influence. Additional PeMS sites were added between 2018 and 2022, so the VMT calculation in all years excluded data from PeMS sites added after January 2018.

Monthly natural gas distribution data were obtained from Pacific Gas & Electric (PG&E).^45^ This data set was reported by postal zip code. Reported natural gas distributed to each zip code was scaled by the fraction of the zip code area within the BEACO_2_N region of influence.

## 3 Results and Discussion

### 3.1 Interannual Trends in CO_2_ Emissions

**Figure 2:**
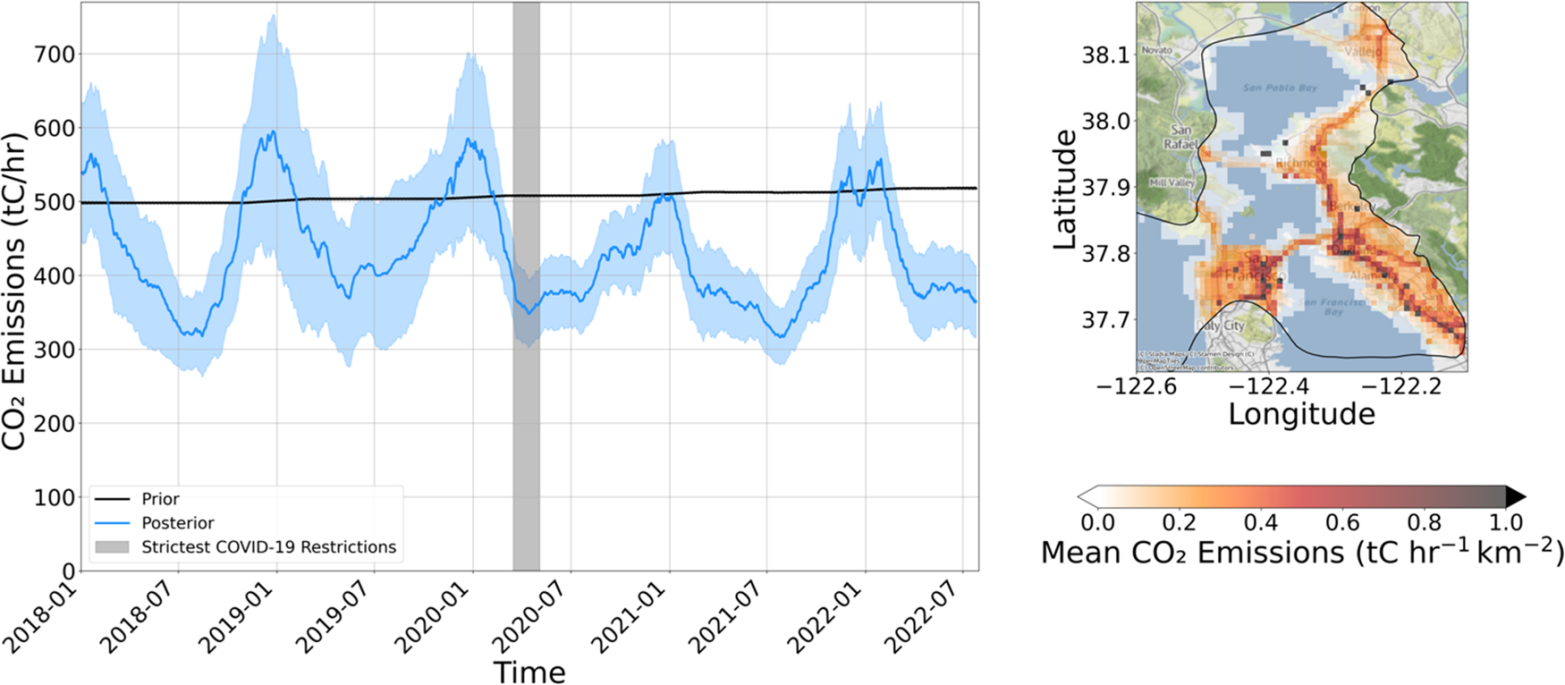
Five years of prior (black) and posterior (blue) anthropogenic emissions in the region of influence (shown on the right). Prior and posterior emissions are rolling 6-week averages. Uncertainty in posterior (derived from surface influence) shown in light blue shading. Period of shelter-in-place order during COVID-19 marked shaded in gray. Background map credits: © Stadia Maps (stadiamaps.com), © Stamen Design (stamen.com), © OpenMapTiles (openmaptiles.org), © OpenStreetMap (openstreetmap.org/copyright).

Solving for posterior emissions for each day within the BEACO_2_N footprint yields the result shown in Figure 2 along with the steadily increasing prior for comparison. Both prior and posterior are shown as a rolling 6-week average, smoothing any diurnal and weekly effects. Emissions shown are anthropogenic emissions only (SIF-GPP-derived biosphere fluxes are subtracted off). While the biospheric uptake derived from SIF-GPP can be strong during daytime in the growing season, on seasonal time scales, the biospheric fluxes derived using SIF-GPP relationships are found to be small relative to the anthropogenic fluxes in the region, and the seasonality does not match the seasonal cycle of the posterior fluxes. Full posterior emissions before the biosphere subtraction are shown in Figure S4. There are larger biospheric fluxes found just outside of the region of influence, but the region of influence itself is quite urban, and the mean biospheric flux across all seasons and times is only −7 tC/h in the region. The relative size of the background, biosphere, and emission contributions to the total CO_2_ concentrations is explored further in Figure S5.

While the prior emissions do not vary seasonally, a notable seasonal cycle is introduced in the posterior, with posterior emissions in the winter running about 40% higher than in the summer. Some of this seasonality could be residual biospheric fluxes not seen in SIF-GPP (although the seasonality does not match the SIF-GPP seasonality). However, we do expect to see seasonal differences in emissions from residential and commercial natural gas heating in the winter, which is used by the majority of buildings in the region.^46^ The Bay Area Air Quality Management District (BAAQMD) reports residential fuel usage as the source of 7% of Bay Area GHG emissions.^34^ However, we expect that some industrial emissions will have a strong seasonal cycle matching that of home heating from the heating of commercial facilities. About half of the observed seasonal trend is explained by the PG&E reported natural gas consumption alone and the seasonality of reported natural gas combustion matches the seasonality of our posterior, as discussed more in the sectoral decomposition (Section 3.3).

The decrease in emissions during the COVID-19 shelter-in-place order is highlighted in Figure 2 (gray-shaded period). Emissions were the lowest in April 2020 of any April, the only full month impacted by the order, which was issued on March 16, 2020 (the strictest restrictions began to be lifted by May 4, 2020). A comparison of April 2019 to April 2020 yields a 13.4% decrease in total emissions (13.3% decrease on weekdays, 13.9% decrease on weekends).

We observe a decreasing emissions trend at a rate of −1.8 ± 0.3%/year from 2018 levels. This trend was computed using ordinary least-squares (OLS) on the hourly data after deseasonalizing the posterior by subtracting the seasonal component of the time series. The seasonal component is calculated by detrending the hourly posterior, computing the mean value of the detrended data for each day of year (mean across the 5 years), and smoothing with a 90-day rolling mean. The uncertainty (0.3%/year) in the trend represents the 99.9% confidence interval in the OLS fitting. The decreasing trend is found to be statistically significant (*p* = 0.0005) using a seasonal Mann–Kendall (MK) trend test on the posterior.^47^

**Figure 3:**
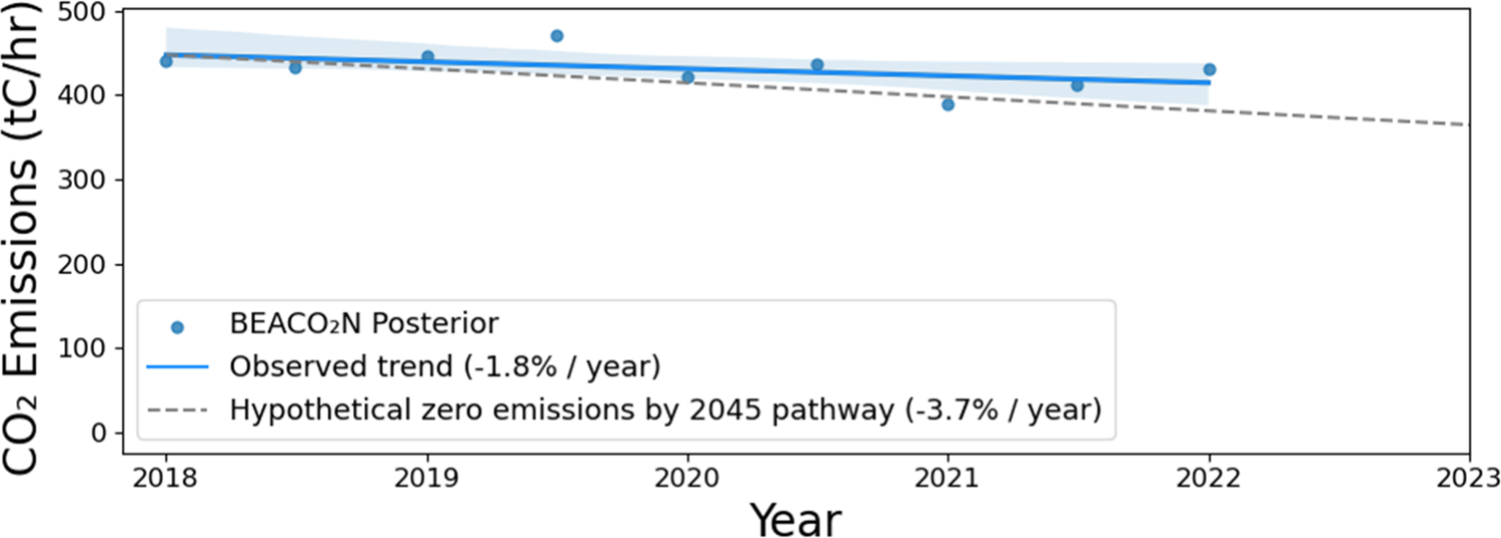
Emissions trend from 2018 to 2022 compared to the rate of emissions decrease required to achieve zero emissions by 2045. Each point represents the average emissions of a 6-month period. Points are plotted on the first day of the 6-month period. Shading depicts 95% confidence interval in linear fit.

We can greatly improve the confidence in the posterior using temporal averaging (see details of this uncertainty analysis in Section 3.2), so we utilize 6-month averaging of the posterior emissions (9 time periods) to visualize the emission trends over our study period. January–June and July–December were averaged in each year. Six-month averaging removes the seasonal cycle of natural gas combustion. A hypothetical zero by 2045 pathway is shown from the beginning of the study period onward. Figure 3 shows the mean posterior result for each of the 6-month periods in the inversion, as well as the fitted −1.8%/year trend. Note that the trend is observed to be −1.8%/year both when fitted on the 6-month averaged data and when fitted on the deseasonalized hourly data.

This result is comparable to the results of other studies in this urban region, although the region of influence studied here is unique. The city of Oakland’s inventory finds a 21% decrease in CO_2_ emissions from 2005 to 2019 (−1.5%/year relative to 2005 levels).^48^ Luqman et al., 2023 reported a −0.7%/year change in CO_2_ emissions in the greater SF Bay Area from 1998 to 2018 (relative to midpoint) using The Open-Data Inventory for Anthropogenic Carbon inventory and taking into account the changing boundaries of the urban region.^49^ The SF Bay Area was one of only 6 urban regions in which Luqman et al. reported a decreasing emissions trend of 91 urban regions investigated.

The current rate of emissions changes (−1.8 ± 0.3%/year), extrapolated to 2045, results in emissions of 225 tC/h (range: 196–264 tC/h) in the year 2045, or about half of 2018 levels. Achieving net-zero emissions by 2045 (as is the goal stated by a number of municipalities in the region of influence) will require an increase in the rate of emissions reductions we observe in this study or the use of substantial carbon offsets and/or capture. Importantly, our analysis is limited to scope 1 emissions, and our inversion does not quantify progress on scope 2 or 3 emissions that occur outside of the region of influence.

### 3.2 Uncertainty Analysis

The posterior error covariance matrix can be used to characterize the uncertainty in large inverse problems; however, computing this matrix is computationally intractable in this case. In lieu of directly constructing the posterior error covariance matrix, we characterize the error in the posterior area indirectly as a function of the footprint surface influence. The changing nature of the network and availability of data (Figure 1) over the 5-year period presents an additional challenge for error characterization. It was important to ascribe heteroskedasticity to the errors, such as to acknowledge the temporally changing network influences. To do this, we examined the posterior emissions as a function of the STILT footprint surface influence. We fit an exponential decay to this function as shown in Figure S6, which shows that this error is strongly inversely correlated with the number of nodes operating in the network. Posterior uncertainties shown in Figure 2 are the sum of the absolute error from surface influence and an estimated 10% error on the posterior. We estimate 10% additional error to have the uncertainty approximately match the standard deviation of the posterior (6-week rolling sigma of the posterior = 105 tC/h). We assume that we cannot account for all sources of error and the error is at least 10% even when surface influence is large enough to show convergence of the posterior. We find that the error in the posterior reduces according to the central limit theorem with the square root of the number of measurements used. Uncertainties are further explored in Figures S7 and S8. Errors are reduced substantially with greater than 4 weeks of averaging in the posterior, likely due to the reduction in meteorological biases from the HRRR product from averaging.

The described method yields an uncertainty of 74 tC/h on the posterior. To derive the prior uncertainties using the same method we use the y-intercept of Figure S6B (because the emissions are the prior when influence is zero) as the absolute error and again add an additional 10% error. This yields an emissions uncertainty of 220 tC/h on the prior. Errors are therefore reduced from the prior to the posterior by 66%.

### 3.3 Trends in Vehicle Fleet Fuel Efficiency

To investigate the changing efficiency of the region’s vehicle fleet, a simple decomposition of the posterior emissions was conducted. It is assumed that the majority of posterior anthropogenic emissions can be roughly decomposed into seasonally unvarying emissions from point sources (i.e., cooking and industrial emissions), emissions with a strong seasonal cycle (i.e., home and commercial heating), and traffic emissions. Electricity generation is not a significant source of direct (scope 1) emissions in the region. The largest electricity-generating power plant in the region of influence is the Dynegy Oakland Power Plant, which reports emissions of only 0.25 tC/h.^34^ We write this decomposition in eq 3:
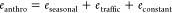
3

We assume that the overall efficiency of the vehicle fleet is changing linearly in time as older, less efficient vehicles are replaced by newer, efficient, and electric vehicles. We assume that the efficiency of natural gas combustion does not change on this time scale and that other point source emissions stayed constant. This yields eq 4:
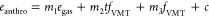
4where *t* is time, *m*_1_*e*_gas_ = *e*_seasonal_, *m*_2_*tf*_VMT_ + *m*_3_*f*_VMT_ = *e*_traffic_, and *c* = *e*_constant_. Two independent data sets, not used to inform the prior inventory, were used for natural gas combustion in the region (*e*_gas_) and vehicle miles traveled in the region (*f*_VMT_). For seasonally varying emissions, monthly natural gas distribution data were obtained from the utility PG&E.^45^ These gas data were converted to emissions using a carbon intensity of 50.3 g CO_2_/MJ for natural gas.^50^ For *f*_VMT_, the PEMS data set was used as a proxy for vehicle miles traveled in the region.^44^ Preprocessing of the two data sets is described in Section 2.7. We conduct a simple multiple linear regression (MLR) to derive the coefficients *m*_1_, *m*_2_, *m*_3_, and *c*, using the posterior derived hourly anthropogenic emissions as ems_anthro_. The value of *m*_1_ was 2.3, which means about half of the observed seasonal trend is explained by the PG&E reported natural gas consumption. The constant emissions (*c* = *e*_constant_) were 156 tC/h. The term *m*_2_*t* + *m*_3_ can be factored out, and the rate at which this value changes over the 5-year study period is a reasonable proxy for the rate of change of overall vehicle fleet efficiency (average CO_2_ emissions per vehicle mile traveled). The fitting is conducted on hourly data, and the *R*^2^ between the simplified emission model predicted emissions and the posterior “true” emissions is 0.44, with mean absolute error of 74 tC/h for the total emissions of the influence region. The mean absolute error is reduced to 44 tC/h once emissions are averaged monthly.

**Figure 4:**
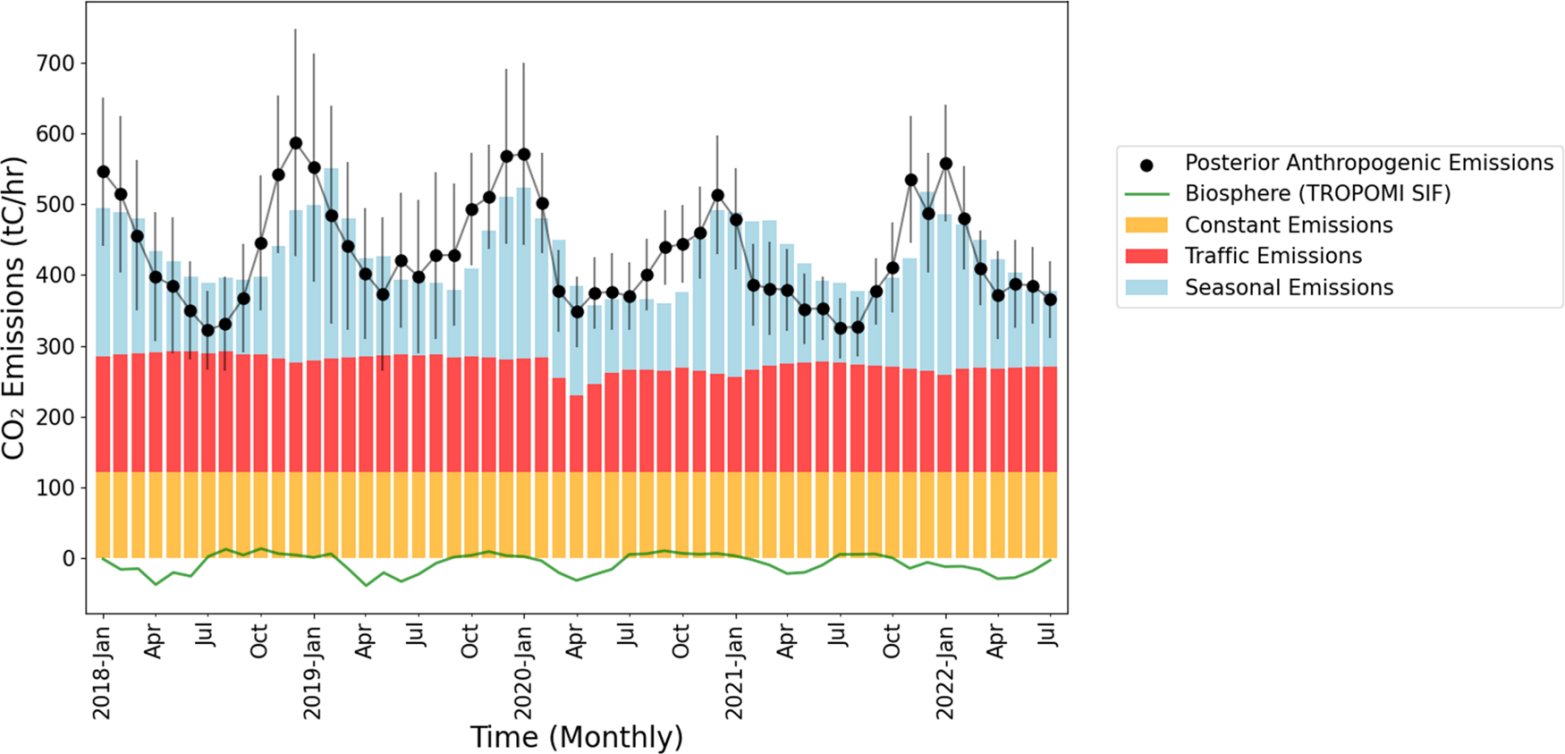
Sectoral decomposition of emissions by multiple linear regression.

Figure 4 shows the result of sectoral decomposition by multiple linear regression. The traffic emissions have minimal interannual or seasonal variability aside from the drop in 2020 during the COVID-19 shelter-in-place order. All seasonality in the posterior is attributed to natural gas combustion after SIF-GPP-derived biosphere fluxes are subtracted off. We also show the magnitude and seasonality of the SIF-GPP-derived biospheric fluxes, which are small compared to the anthropogenic fluxes in the region. In winter 2019, there is a 2-month lag between the maximum posterior and the maximum natural gas usage. Winter 2019 is one of the most uncertain periods for the posterior. In 2020, 2021, and 2022, the maximum posterior occurs in the same month (±1 month) as maximum natural gas usage. Differences between the MLR emissions and posterior anthropogenic emissions may result from unresolved seasonal biosphere (not captured by SIF) or from anthropogenic emissions for which these 3 emissions categories are not reliable proxies.

Solving for the coefficients in eq 4 yields the result that the overall vehicle fleet CO_2_ efficiency (emissions per mile traveled) improved by 11.9% over the nearly 5-year study period, or 2.6%/year. This result is sensitive to anthropogenic/biogenic partitioning; hence, better constraints on urban biospheric fluxes will be important for application of the method. We tested values of the biosphere in the range of 0.5–1.5× SIF-GPP derived values for the partitioning to get uncertainty bounds on the emissions reduction rate of 2.6 ± 0.7%/year. This result is comparable to a previous finding (using a simplified model to interpret the BEACO_2_N observations) of a 7.6% improvement in vehicle fuel efficiency over the 3-year period from 2018 through 2020^51^ and indicates the trend of improved vehicle efficiency continuing into 2021–2022. Our result closely matches the California Air Resources Board’s Emissions Factors Model (EMFAC) model from 2017, which predicted a 2.5%/year improvement in overall vehicle fleet CO_2_ efficiency for the Bay Area from 2018 to 2022.^52^ This 11.9% improvement in overall vehicle fleet efficiency likely results from a combination of adoption of electric vehicles and hybrids as well as the gradual retirement of the oldest and least efficient vehicles in the fleet. We show in Figure S10 that low-emitting and zero-emission vehicles are being adopted more rapidly in the BEACO_2_N region of influence than in the state of California as a whole according to vehicle registration data obtained from the California Department of Motor Vehicles.^53^ As of January 2022, plug-in hybrid, battery electric, and hydrogen fuel cell vehicles make up 4.2% of the fleet in our region of influence (up from 2.1% in October 2018). For the state of California, these vehicle classes made up only 2.7% of the fleet in January 2022 (1.4% in October 2018). As such, we do not expect that the rate of emission decreases we report here for the SF Bay Area are representative of the entirety of the state of California.

In this study, we have presented CO_2_ emissions for a region of the San Francisco Bay Area as constrained by observations from the BEACO_2_N network, the HRRR-STILT model, and Bayesian inverse modeling from 2018 to 2022. We find that CO_2_ emissions in the BEACO_2_N region are decreasing at a rate of 1.8 ± 0.3%/year from 2018 levels. Despite this progress, a projected continuation of these emission reductions only leads to a 50% reduction of 2018-level emissions by 2045, falling short of the ambitious zero-emission targets set by numerous cities in the region. Sectoral decomposition of the posterior by multiple linear regression allows us to calculate the rate of change of the fleetwide CO_2_ emission factors, which we find to be −2.6 ± 0.7%/year. This study advances the field of urban interannual CO_2_ inversions, providing an example of an effective top-down methodology for carbon monitoring and management at the city scale. Our findings emphasize the urgent need for accelerated climate policy and action to achieve the ambitious zero-emission targets cities seek.

## Data Availability

BEACO_2_N data are available at beacon.berkeley.edu. STILT is available from https://uataq.github.io/stilt/#/. Inversion and analysis code shared upon request.

## Supporting Information Available

Detailed description of the methods of the inversion system, table of parameters used in the inversion, process flow diagram, cumulative influence contours, emissions including biosphere contribution, relative contributions to observed concentrations, further descriptions of uncertainty quantifications, background concentrations, and vehicle registrations (PDF)
