# Supplementary material for: Sustained Reductions of Bay Area CO2 Emissions 2018–2022
Source: Environ Sci Technol. 2024 Apr 4;58(15):6586–94. doi: 10.1021/acs.est.3c09642 (PMC11025126; doi:10.1021/acs.est.3c09642)
Supplement: Supplementary file 1 — es3c09642_si_001.pdf [file es3c09642_si_001.pdf]

Supporting Information

Sustained Reductions of Bay Area CO_2 Emissions 2018-2022

Naomi G. Asimow*[1], Alexander J. Turner[1], and Ronald C. Cohen*[1,2]

[1]Department of Earth and Planetary Science, University of California, Berkeley, Berkeley, CA 94720, USA

[2]College of Chemistry, University of California, Berkeley, Berkeley, CA 94720, USA

Correspondence to: Naomi Asimow (nasimow@berkeley.edu) and Ronald C. Cohen (rccohen@berkeley.edu)

This file includes:

Supplementary Text S1 to S2

Supplementary Table S1

Supplementary Figures S1 to S10

Number of Pages: 12

Text S1: Details on the construction and design of the inversion system

We invert for both the fluxes within the specified domain and the background concentration at the domain edge according to:

$$\hat{\mathbf{x}} = \mathbf{x}_a + (\mathbf{HB})^T(\mathbf{HBH}^T + \mathbf{R})^{-1}(\mathbf{y} - \mathbf{Hx_a})$$

where $\hat{\mathbf{x}}$ is a vector of the expected posterior fluxes at each hour and grid-cell, and the background concentrations at each hour and each of the four domain edges. $\mathbf{x_a}$ is the prior value of the fluxes (emissions inventory) and the prior background concentrations, **H** is the operator that connects the observations to emissions, combining the HRRR-STILT footprints and indicator values (0 or 1) for which background concentration to use, **B** is a prior error covariance matrix, **R** is the model-data mismatch error covariance matrix, **y** is the BEACO$_2$N measurements. This equation is derived from assuming Gaussian distributions of errors and solving for the probability density function P(**x**|**y**) where $\hat{\mathbf{x}}$ is the expected value of the probability density function.[1] For computational efficiency we express the prior error covariance matrix **B** as a Kronecker product of a spatial prior error covariance matrix and a temporal prior error covariance matrix, as described by Yadav and Michalak.[2]

The inversion framework is similar to the method described in Turner et al.[3] but has been modified to solve dynamically for background concentrations. To decrease the impact of uncertainty in background concentrations on the result, we add elements to the state vector ($\mathbf{x}$) for background concentrations at the four borders of the domain at every hour, similar to the method of Henne et al.[4] For each observation, the footprint was used to determine which edge the parcel had traveled from and the corresponding prior background. No time offset was used (i.e., each observation was assigned a simultaneous GEOS background) in order to avoid introducing errors from the PBLH changing during the period the parcel transverses the domain.[5] Each row of the H matrix thus contains all footprint elements for a particular observation, as well as zeros for all but the selected background side and time such that $\mathbf{H}\boldsymbol{x_a}$ gives the prior predicted concentration for each observation as computed from the footprints, prior emissions, and prior background concentration. $\mathbf{Hx}_a$ is constructed as:

$$\mathbf{Hx}_a = \begin{bmatrix} \frac{\partial y_1}{\partial x_{(1,1,1)}} & \frac{\partial y_1}{\partial x_{(1,2,1)}} & \cdots & \frac{\partial y_1}{\partial x_{(m_x,m_y,1)}} & \mathbb{1}(y_1, b_{(N,t=1)}) & \cdots & \mathbb{1}(y_1, b_{(S,t=1)}) & \frac{\partial y_1}{\partial x_{(1,1,2)}} & \cdots & \mathbb{1}(y_1, b_{(S,t=m_t)}) \\ \frac{\partial y_2}{\partial x_{(1,1,1)}} & \frac{\partial y_2}{\partial x_{(1,2,1)}} & \cdots & \frac{\partial y_2}{\partial x_{(m_x,m_y,1)}} & \mathbb{1}(y_2, b_{(N,t=1)}) & \cdots & \mathbb{1}(y_2, b_{(S,t=1)}) & \frac{\partial y_2}{\partial x_{(1,1,2)}} & \cdots & \mathbb{1}(y_2, b_{(S,t=m_t)}) \\ \vdots & \vdots & \ddots & \vdots & \vdots & \ddots & \vdots & \vdots & \ddots & \vdots \\ \frac{\partial y_n}{\partial x_{(1,1,1)}} & \frac{\partial y_n}{\partial x_{(1,2,1)}} & \cdots & \frac{\partial y_n}{\partial x_{(m_x,m_y,1)}} & \mathbb{1}(y_n, b_{(N,t=1)}) & \cdots & \mathbb{1}(y_n, b_{(S,t=1)}) & \frac{\partial y_2}{\partial x_{(1,1,2)}} & \cdots & \mathbb{1}(y_n, b_{(S,t=m_t)}) \end{bmatrix} \begin{bmatrix} x_{(1,1,1)} \\ x_{(1,2,1)} \\ \vdots \\ x_{(m_x,m_y,1)} \\ b_{(N,t=1)} \\ \vdots \\ b_{(S,t=1)} \\ x_{(1,1,2)} \\ \vdots \\ b_{(S,t=m_t)} \end{bmatrix}$$

where each $\partial y_i / \partial x_{(j,k,t)}$ element is the footprint influence on an observation y_i within the column vector $\mathbf{y} = [y_1 \ldots y_n]$ for an emission in the prior emission vector ($\mathbf{x_a}$) from a particular pixel in space (j,k) and in time (t). The footprints for emissions for one emission time occupy $m_x m_y$ columns. Between footprints for each time are columns including the background indicator functions $\mathbb{1}(y_i, b_{(side,t)})$ for a particular time, where side is the cardinal direction (N/E/W/S)

and $b_{(side,t)}$ is the prior value of the background concentration at one edge of the domain and time. The indicator function $\mathbb{1}(y_i, b_{(side,t)})$ always equals 0 except for the corresponding side and time for a given observation y_i, when the indicator function evaluates to 1. $\mathbf{x_a}$ contains the prior emissions $x_{(j,k,t)}$ at each grid point (indexed j,k) and each time t. After the emissions for each time t there are 4 values for the 4 prior background concentrations at the time t at each of the four sides of the domain, b(side,t). Here we invert over a 157 x 127 km domain for 96 hours at a time (24 hours each day + 36 hours before and after), so $m_x = 157$, $m_y = 127$, and $m_t = 96$. $\mathbf{x_a}$ has dimensions m x 1 and **H** has dimensions n x m, where $m = (m_x m_y + 4)\ m_t$.

B is expressed as a Kronecker product of a spatial prior error covariance matrix and a temporal prior error covariance matrix. The mixed units (fluxes and backgrounds) in the spatial prior error covariance matrix require individual treatment. Fluxes were treated identically to the method described in Turner et al. 2020.[3] Each matrix element represents the product of the correlation of the prior fluxes of two spatial pixels with the geometric mean of the magnitudes of the fluxes of the two pixels, scaled by their distance using a spatial decay scale of 5 km. Elements for the background represent the product of the geometric mean of the backgrounds, a correlation of 1 or 0.1 (for diagonals or off-diagonals, respectively), and an additional scaling factor of 1×10^{-4}. The choice of the scaling on the backgrounds was somewhat arbitrary, but was determined after several iterations to be a reasonable value that allows the backgrounds to change within a few ppm (see Figure S9), giving sufficient flexibility for the fluxes. Elements in the matrix block relating backgrounds and fluxes were all set to 0. The temporal prior error covariance matrix assumes a 5 hour temporal decay timescale, which is applied based on the number of hours apart regardless of day (such that 6 am on Monday is closely correlated to 6 am on Tuesday). It also assumes a 24 hour temporal decay timescale, such that correlations decrease over time. The Kronecker decomposition was limiting in this case because it did not allow us to impose different temporal decay parameters on the fluxes and backgrounds. However, given the close connection of background concentrations with urban fluxes outside of the inversion domain, it is logical for concentrations entering the domain to vary on similar time scales to emissions.

The model-data mismatch error covariance matrix, **R**, combines the additive contributions of instrument error, model error, and background error. For instrument error we use the standard deviation of the sub-hourly measurements (with a minimum of 1 ppm). The time of day dependent model errors were modified from the values in Turner et al. 2020 to more closely match the observation-prior mismatch, giving less weight to observations at night when planetary boundary layer height (PBLH) is thinnest and most uncertain. This change corrects a time zone conversion bug in the previous study. Correction of this error was found to have minimal impact on the results. These model errors may be an underestimate due to the high level of uncertainty in the HRRR wind field. Comparison of the HRRR wind field to 1 year of observational data at about ~200 observational sites showed large uncertainties at individual sites, though the regional mean hourly wind speeds agreed well between the model and observations (R^2=0.87). For background errors we used the standard deviation of the prior backgrounds at each time. We impose a concentration correlation length scale of 2 km and temporal decay scale of 1 hour, as described in Turner et al. 2020.[3] Each element of the matrix is the product of the geometric mean of the total error (instrument error, model error, and background error)

for two observations, scaled by their distance using a spatial decay scale of 2 km and their time difference with a decay scale of 1 hour.

We generate posterior fluxes once for each day of the study period using 96 hour overlapping windows. Each daily inversion uses the 24 hours of interest plus the 36 hours before and after that day. This allows observations just outside of a 24 hour window to inform emissions during that day. Emissions estimates for the 36 hour buffers are discarded and the daily posterior estimates for each 24 hour period are used for analysis. The computational cost of each 96 hours is substantial due to the size of the matrixes involved. Each 96 hour inversion was run on an Intel Xeon Skylake 6130 @ 2.1 GHz node on the UC Berkeley Savio cluster with 32 cores/node and memory of 384 GB/node. Typical runtime to compute the posterior for one 96 hour inversion was around 10 hours.

Text S2: Determination of the Influence Region

Computation of the diagonal of the averaging kernel matrix is one method to define the region of interest, but this matrix has dimension m x m and thus computing it directly is typically computationally intractable. In lieu of constructing the full matrix, we calculate the cumulative influence of the footprints in the region and define the top 40th percentile as the region to which we are sensitive (influence region).

Turner et al., 2020 evaluated different cumulative influence contours and found that the 40th percentile cumulative influence contour encapsulates most of the posterior changes.[3] Other cumulative influence contours are presented in Figure S2. Outside of this region, there is usually not enough surface influence to make a significant update on the prior, so posterior emissions strongly resemble the prior emissions. While the region of influence does change slightly depending on which BEACO_2N nodes are operational in a given timeframe and the particular meteorology of that timeframe, one region of influence, representing the mean, is used throughout this analysis. The seasonality of the region of influence is explored in Figure S3. In all seasons the majority of trajectories enter the domain from the west, traveling via the Golden Gate (the straight famous for the Golden Gate Bridge, which connects the Pacific Ocean to the SF Bay). However in winter there is an increase in the influence of the northeast area of the domain, as trajectories from that direction are more common during winter than other seasons. The “overall” contour used excluded this region to which we are only sensitive in winter. The majority of the (land) area for the overall 40% contour also appears within land areas of the 40% contours of each season, indicating that we are sensitive to the overall region of influence throughout the year.

Table S1: Inversion parameters

Parameter	Value
Spatial resolution of **x**	1 km ($m_x = 157$, $m_y = 127$)
Temporal resolution of **x**	1 hour ($m_t = 96$)
Length of **x**	$m = (m_x m_y + 4)\ m_t = 1{,}914{,}528$
Back trajectory model for **H**	STILT
Footprint resolution in **H**	1 km, hourly
Meteorology for back trajectory model	HRRR
Meteorological resolution	3km, hourly
Decorrelation length in **B**	5 km
Decorrelation time in **B**	5 h, 24 h
Instrument error in **R**	1σ of sub-hourly measurements (1 ppm minimum error)
Background error in **R**	1σ of N/E/W/S backgrounds
Decorrelation length of observations in **R**	2 km
Decorrelation time of observations in **R**	1 h
Hour of day dependent model errors in **R** (for hours 0 – 23, local time)	[3, 3, 3, 3, 3, 3, 4, 5, 8, 6, 4, 2, 1, 1, 1, 1, 2, 4, 6, 8, 5, 4, 3, 3] ppm

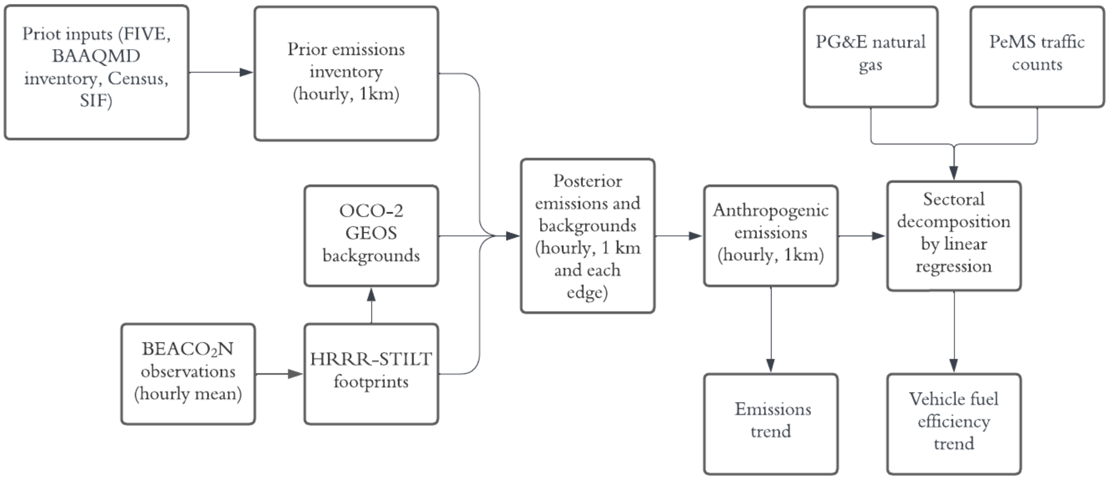

Figure S1: Process flow diagram for the various methods and datasets used in the analysis.

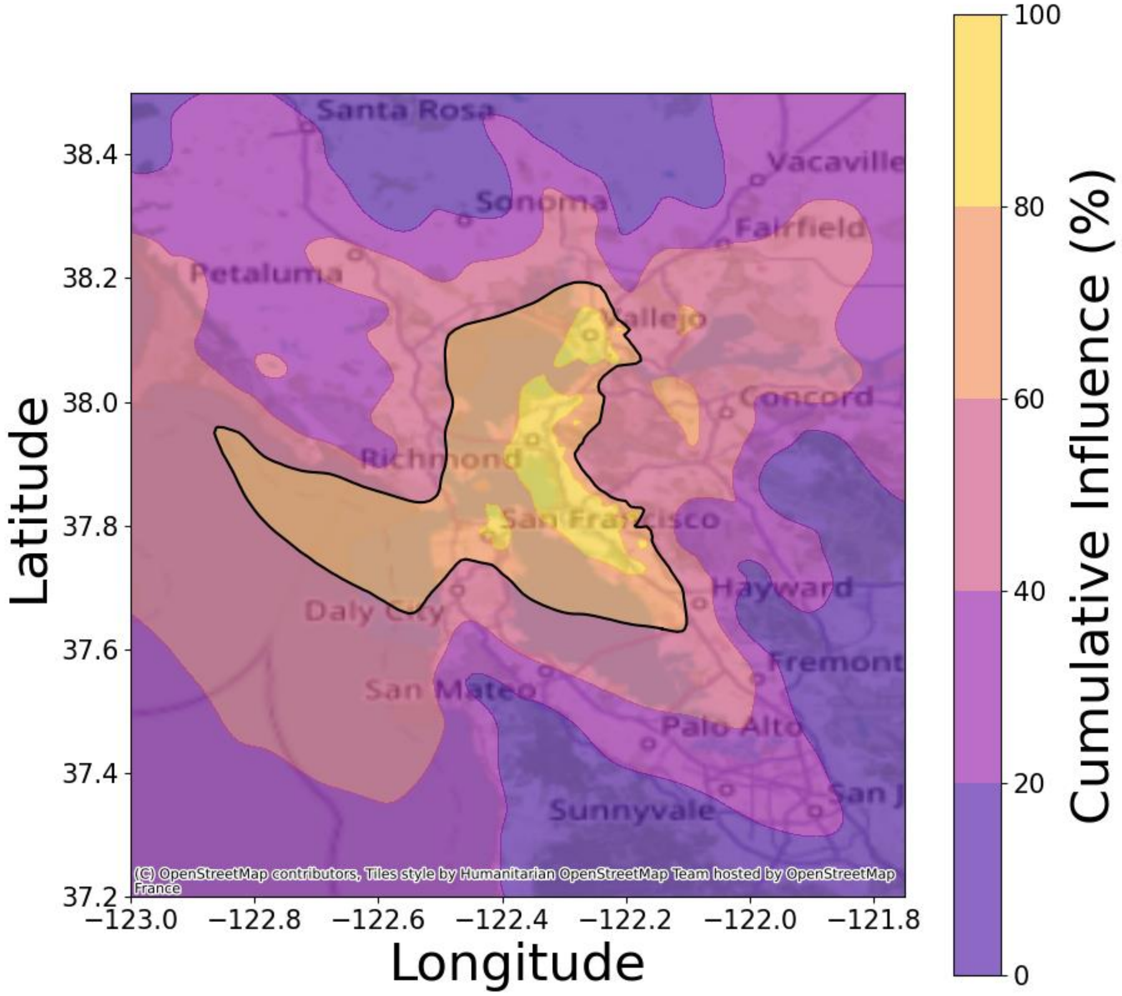

Figure S2: Cumulative influence contours from STILT footprints, normalized to 100%. 40% contour is highlighted in black. Background map credits: © OpenStreetMap contributers.

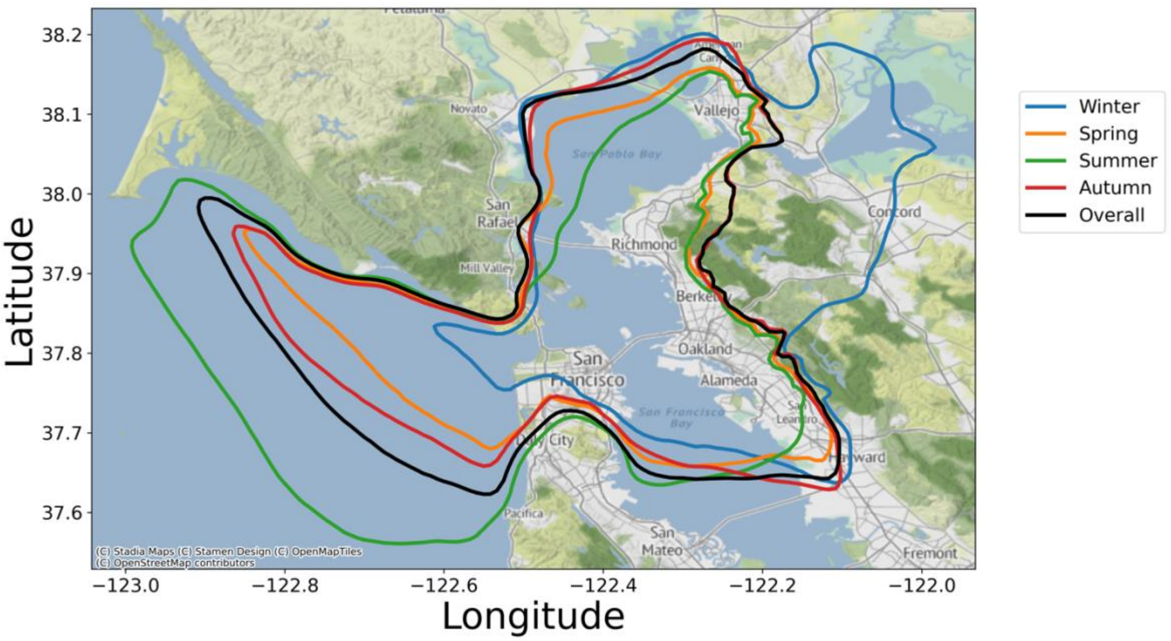

Figure S3: 40% influence contours by time of year: Winter (DJF), Spring (MAM), Summer (JJA), and Autumn (SON), as well as the overall 40% contour for the entire study period (black). Background map credits: © Stadia Maps (stadiamaps.com), © Stamen Design (stamen.com), © OpenMapTiles (openmaptiles.org), © OpenStreetMap (openstreetmap.org/copyright).

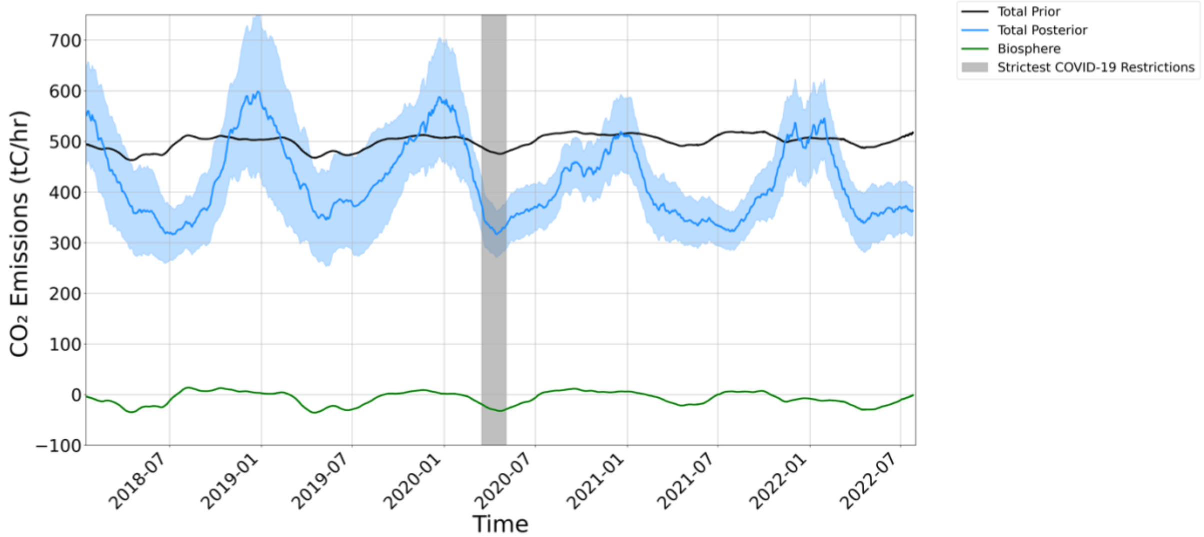

Figure S4: Prior (black) and posterior (blue) total (anthropogenic and biogenic) emissions and the SIF-GPP derived biosphere (green) in region of influence. The prior and posterior shown here are total fluxes before the biosphere is subtracted to get the anthropogenic fluxes used for analysis in the main text. Figure 2 in the main text is the result of subtracting the green line from the blue and black lines shown here. Prior, posterior, and biosphere emissions are

rolling 6-week averages. The uncertainty in the posterior (derived from surface influences plus 10% additional error) is shown in light blue shading. Period of shelter-in-place order during COVID-19 marked shaded in grey.

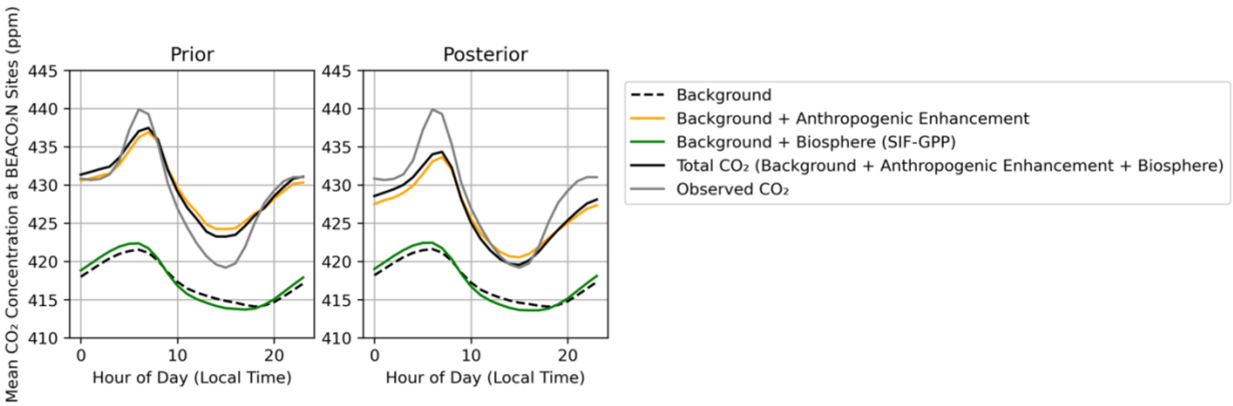

Figure S5: Mean diurnal cycle of the contributions to total concentrations at BEACO_2N sites for the prior (left) and posterior (right) backgrounds and emissions. The observed CO_2 (grey) and SIF-GPP constrained biosphere contributions are the same on the left and right plots. In general, the biosphere contribution is small compared to anthropogenic contribution and the anthropogenic contribution is small compared to background. Mean observed concentrations are higher than the prior predicted in the morning and lower in the afternoon (though this varies seasonally). The mean posterior total resembles the observed concentrations in the well-mixed, high PBLH afternoon, when the model errors are smallest. The mean morning posterior total concentrations were also decreased relative to the prior morning total concentrations due to relatively long (5 hour) decay timescale used in the prior error covariance matrix and high uncertainty in the model in the morning when PBLH is small and poorly constrained.

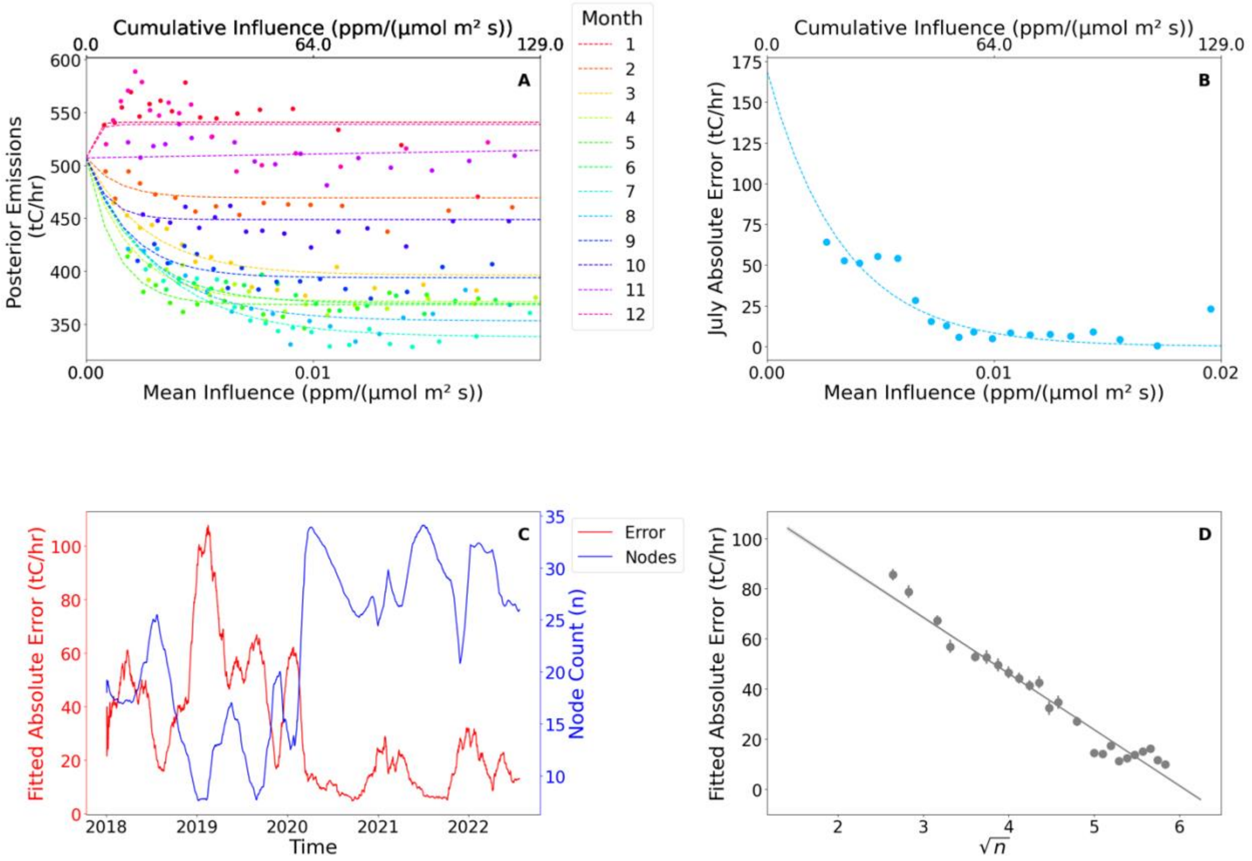

Figure S6: A: Posterior emissions change exponentially with surface influence, converging when the mean footprint influence in the region of influence is greater than 0.01 ppm/μmol/m2/s. This convergence is more pronounced in the summer months, when influence is be larger. Scatter points in A are 20 quantile bins with each point representing the mean of 5% of the hours in a month. Mean influence is the mean surface influence of all the grid cells in each quantile bin. B: July is the month in which the converged emissions differ the most from the prior, so July emissions are used as an upper bound on absolute error, where the convergence value is treated as the true value. C: The error derived from this fitting (red line on plot C) is closely anti-correlated with the number of nodes in operation (blue line on plot C). D: Fitted absolute error reduces linearly with the square root of the number of nodes in operation. Scatter points are 30 quantile bins for the number of nodes.

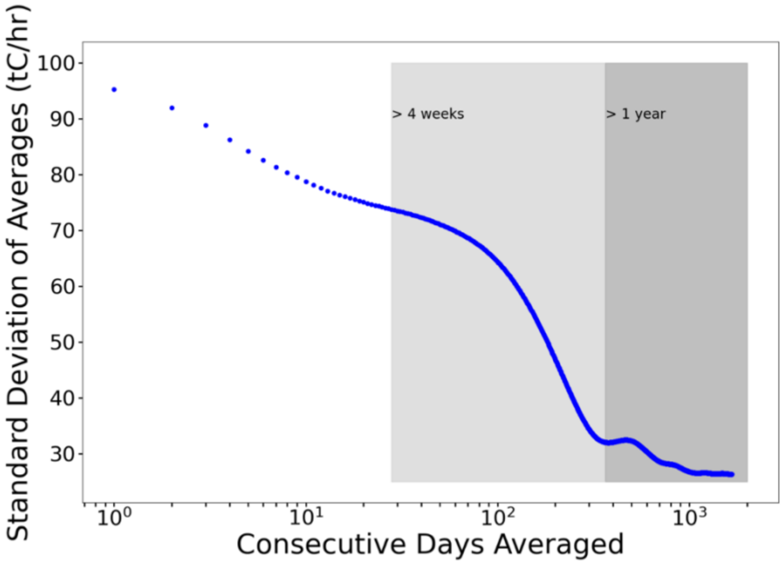

Figure S7: A rolling average of the posterior emissions was calculated, with averaging window on the horizontal axis. The standard-deviation of the averages is shown on the vertical axis. Note the log scale on the horizontal axis. Two convergences are observed at ~4 weeks and ~1 year, highlighted in gray.

We show that standard deviations converge with approximately 4 weeks averaging, and again with approximately 1-year averaging. The convergence at 4 weeks indicates that this is sufficient averaging to minimize the noise from metrological error on the result. The convergence after 1-year is the result of averaging long enough to include the effects of the strong seasonal cycle of posterior emissions.

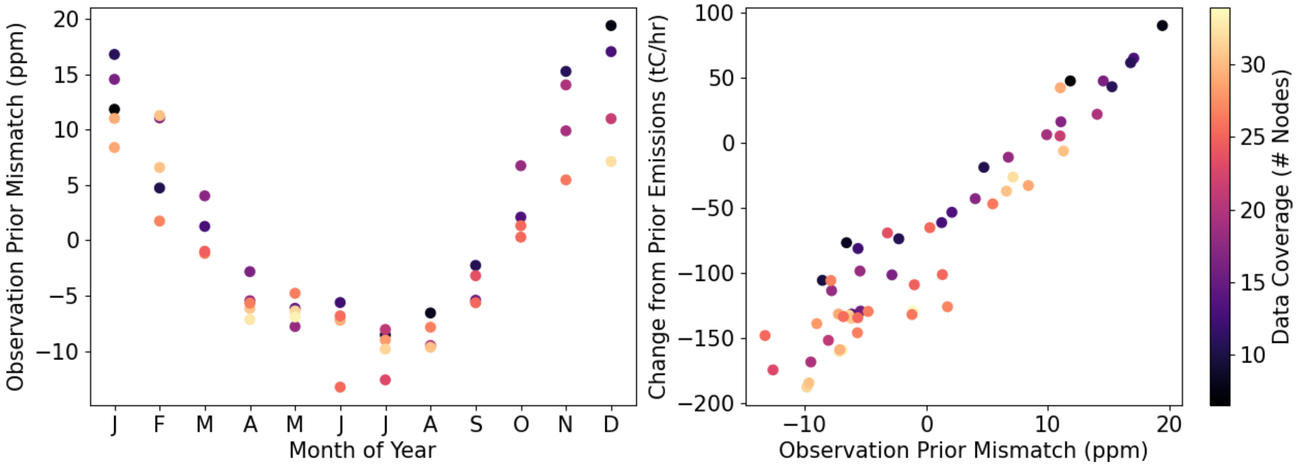

Figure S8: Left: observation prior mismatch ($y\text{-}Hx_a$) by month of year. Right: change in emissions (posterior – prior) by observation prior mismatch. Both plots: Each scatter point represents the average value of a 1-month period and the number of nodes operating that month is indicated with the color of the points.

Here we explore observation prior mismatch (*y-Hxa*), which is the difference between the observed concentrations at the BEACO2N sites and the prior predicted concentrations at the BEACO2N sites from the footprints and prior emissions inventory. We show that there is strong seasonality in the observation prior mismatch. This leads to the strong seasonality in the change from the posterior to the prior emissions (Figure S8, right).

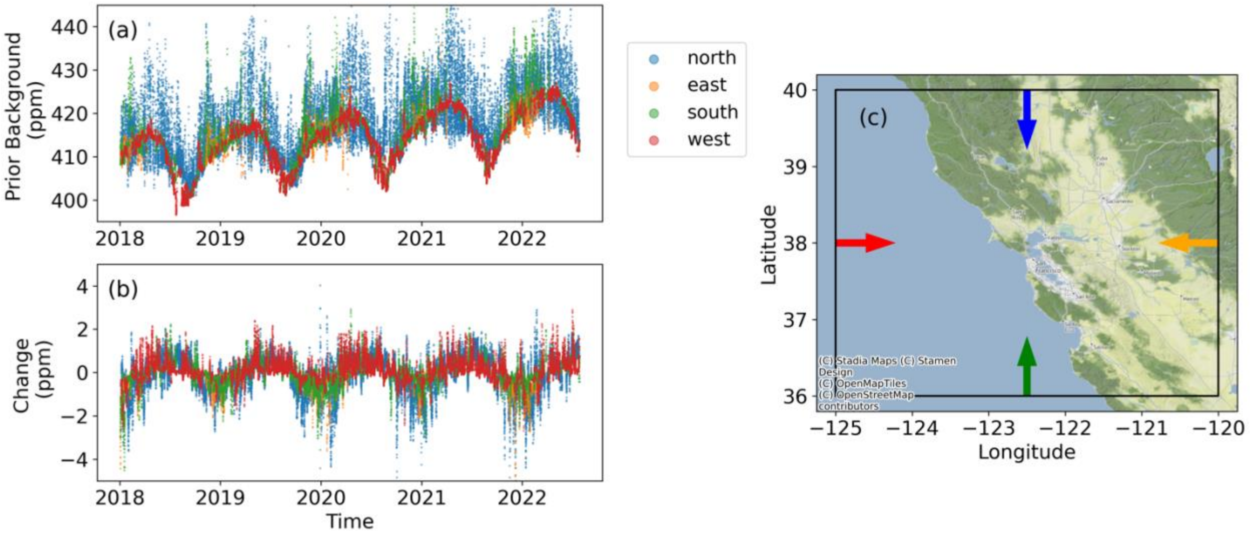

Figure S9: Five years of upwind concentrations at the four domain edges. Prior concentrations from OCO-2 GEOS in (a). Change (Posterior – prior) in (b). (c) shows the locations of the OCO-2 GEOS pixels used for the prior. Background map credits: © Stadia Maps (stadiamaps.com), © Stamen Design (stamen.com), © OpenMapTiles (openmaptiles.org), © OpenStreetMap (openstreetmap.org/copyright).

Inclusion of hourly background concentrations for trajectories entering the domain from the four edges of the domain into the state vector and allowing the inversion to solve for the background concentrations yields the result in Figure S9. Figure S9a shows the prior concentrations from OCO-2 GEOS assimilated dataset. The background to use for each corresponding STILT footprint was determined by the footprints proximity to each of the four edge

centers shown in S9c. The northern edge had a strong diurnal cycle in the backgrounds from OCO-2 GEOS not seen in the other 3 edges. This most likely results from the greater diurnal activity of the PBL over land, as well as the predominate wind direction carrying anthropogenic emissions from The Bay towards the northeast. The change in background concentrations from the inversion (posterior background – prior background) is shown in S9b. We note the slight shift in seasonality between S9a and S9b.

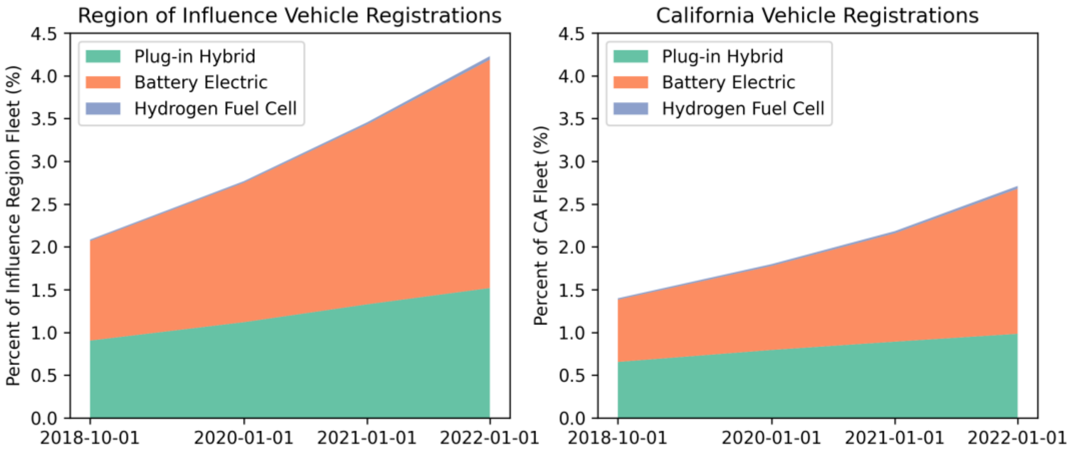

Figure S10: Low-emission and zero-emission vehicles as a percentage of total vehicle registrations within the BEACO_2N region of influence (left) and the state of California (right).

Data obtained from California DMV vehicle registrations reveal that adoption of low-carbon vehicles has been more rapid in the BEACO_2N region of influence than in the state of California as a whole (California Open Data, 2023). The dataset is provided via zipcode. For zipcodes partially contained within the BEACO_2N region of influence vehicle counts were scaled by the fractional area within the region of influence.

References

(1) Rodgers, C. D. *Inverse Methods for Atmospheric Sounding: Theory and Practice*; Series on Atmospheric, Oceanic and Planetary Physics; WORLD SCIENTIFIC, 2000; Vol. 2. https://doi.org/10.1142/3171.

(2) Yadav, V.; Michalak, A. M. Improving Computational Efficiency in Large Linear Inverse Problems: An Example from Carbon Dioxide Flux Estimation. *Geoscientific Model Development* **2013**, *6* (3), 583–590. https://doi.org/10.5194/gmd-6-583-2013.

(3) Turner, A. J.; Kim, J.; Fitzmaurice, H.; Newman, C.; Worthington, K.; Chan, K.; Wooldridge, P. J.; Köehler, P.; Frankenberg, C.; Cohen, R. C. Observed Impacts of COVID-19 on Urban CO2 Emissions. *Geophysical Research Letters* **2020**, *47* (22), e2020GL090037. https://doi.org/10.1029/2020GL090037.

(4) Henne, S.; Brunner, D.; Oney, B.; Leuenberger, M.; Eugster, W.; Bamberger, I.; Meinhardt, F.; Steinbacher, M.; Emmenegger, L. Validation of the Swiss Methane Emission Inventory by Atmospheric Observations and Inverse Modelling. *Atmos. Chem. Phys.* **2016**, *16* (6), 3683–3710. https://doi.org/10.5194/acp-16-3683-2016.

(5) Turnbull, J. C.; Sweeney, C.; Karion, A.; Newberger, T.; Lehman, S. J.; Tans, P. P.; Davis, K. J.; Lauvaux, T.; Miles, N. L.; Richardson, S. J.; Cambaliza, M. O.; Shepson, P. B.; Gurney, K.; Patarasuk, R.; Razlivanov, I. Toward Quantification and Source Sector Identification of Fossil Fuel CO2 Emissions from an Urban Area: Results from the INFLUX Experiment. *Journal of Geophysical Research: Atmospheres* **2015**, *120* (1), 292–312. https://doi.org/10.1002/2014JD022555.

(6) *Vehicle Fuel Type Count by Zip Code - California Open Data*. https://data.ca.gov/dataset/vehicle-fuel-type-count-by-zip-code (accessed 2023-11-15).
